# Cut or Count? Evaluating Advanced Fibrosis Assessment Tools in MASH and Chronic Viral Hepatitis

**DOI:** 10.3390/biomedicines14050988

**Published:** 2026-04-25

**Authors:** Ivana Milošević, Branko Beronja, Nada Tomanović, Marina Đelić, Nikola Mitrović, Dragana Kalajanović, Ankica Vujović

**Affiliations:** 1 Clinic for Infectious and Tropical Diseases, University Clinical Center of Serbia, Bulevar Oslobođenja 16, 11000 Belgrade, Serbia; ivana.milosevic@med.bg.ac.rs (I.M.); brankoberonj99@gmail.com (B.B.); nikolabmi@gmail.com (N.M.); kalajanovicev@gmail.com (D.K.); 2 Faculty of Medicine, University of Belgrade, 11000 Belgrade, Serbia; 3 Institute of Pathology, 1 Dr. Subotica Street, 11000 Belgrade, Serbia; nada.tomanovic@med.bg.ac.rs; 4 Institute of Medical Physiology “Rihard Burijan”, 11000 Belgrade, Serbia; mdjelic011@gmail.com

**Keywords:** chronic viral hepatitis, metabolic dysfunction-associated steatohepatitis, non-invasive tests

## Abstract

**Background/Objectives**: Chronic liver diseases, including metabolic dysfunction-associated steatohepatitis (MASH) and chronic viral hepatitis (CVH), are major global health concerns due to their potential progression to cirrhosis, liver failure, and hepatocellular carcinoma. Because liver biopsy, despite meeting the diagnostic gold standard, is invasive and associated with complications, non-invasive fibrosis assessment tools have been increasingly recommended in clinical practice. This study aimed to compare the diagnostic performance of several non-invasive fibrosis markers (ARR, APRI, FI, FIB-4, API, NFS, BARD) and transient elastography in detecting advanced liver fibrosis (F4) in patients with MASH and CVH. **Methods**: This retrospective study included 237 adult patients (77 MASH, 160 CVH) who underwent liver biopsy between 2017 and 2025 at the University Clinical Center of Serbia. CVH included chronic hepatitis B (CHB) and C (CHC). Patients were evaluated using serum fibrosis indices and TE, and results were compared to histological staging (F0–F4). ROC analysis assessed diagnostic performance. **Results**: Cirrhosis (F4) was more common in CVH than MASH (*p* < 0.001). In MASH, NFS (AUROC 0.931), FIB-4 (0.915), BARD (0.872), and APRI (0.878) showed high diagnostic accuracy for F4. In CHC, APRI (0.931), FIB-4 (0.863), and TE (0.938) had strong performance, while in CHB, TE (0.987) outperformed FIB-4 (0.821). Sensitivity and specificity varied by test and cohort, with TE consistently yielding the best results where available. **Conclusions**: Non-invasive methods, particularly NFS and FIB-4 for MASH and TE for CVH, effectively identify advanced fibrosis. Their application could significantly reduce the need for biopsy, especially in high-risk groups. TE demonstrated superior accuracy, but access limitations highlight the continued relevance of serum-based scores.

## 1. Introduction

Chronic liver diseases, including metabolic dysfunction-associated steatohepatitis (MASH) and chronic viral hepatitis (CVH), represent a major global health burden due to their potential progression to cirrhosis, liver failure, and hepatocellular carcinoma (HCC) [1,2,3]. Accurate staging of liver fibrosis is essential for assessing disease severity, guiding clinical management, and determining prognosis [4]. Although liver biopsy remains the gold standard for fibrosis assessment, its invasive nature, risk of complications, and limited feasibility—particularly in resource-constrained settings—have led to increasing reliance on non-invasive approaches [5,6,7]. Consequently, major hepatology guidelines (EASL, AASLD) recommend the use of non-invasive methods for fibrosis evaluation [8,9,10,11,12,13].

A range of non-invasive tools, including serum-based indices and elastography, has been developed and validated across different patient populations [14,15]. Commonly used serum markers include APRI, FIB-4, NFS, ARR, FI, API, and BARD scores, while transient elastography (FibroScan^®^) represents a widely used imaging-based modality [16,17,18,19,20,21,22]. These methods are less invasive, more accessible, and cost-effective, making them suitable for routine clinical practice.

This retrospective single-center study aims to compare the diagnostic performance of ARR, APRI, FI, FIB-4, API, NFS, and BARD scores, as well as transient elastography (TE) in detecting advanced liver fibrosis (F4 stage) among patients with MASH and CVH, two of the most common causes of chronic liver disease globally. By evaluating their sensitivity, specificity, and accuracy, the study addresses the potential of these non-invasive tools to support timely clinical decisions in everyday practice.

## 2. Materials and Methods

This retrospective observational study was conducted at the Clinic for Infectious and Tropical Diseases of the University Clinical Center of Serbia in Belgrade, the largest national center for the treatment of viral hepatitis. The study included 237 adult patients who underwent blind percutaneous liver biopsy between 2017 and 2025 as part of routine diagnostic workup for the etiology and staging of CHV. Based on clinical, serological, and histological findings, patients were categorized into two main cohorts: MASH (n = 77, 32.5%) and CVH (n = 160, 67.5%). The CVH cohort was further subdivided into chronic hepatitis C (CHC; 58.7%) and chronic hepatitis B (CHB; 41.3%) subcohorts.

MASH was diagnosed in patients exhibiting hepatic steatosis and lobular inflammation on liver biopsy, following the exclusion of significant alcohol consumption, defined as >210 g/week in men or >140 g/week in women; chronic infections with hepatitis B or C virus; HIV infection; drug-induced steatosis; and other concomitant chronic liver diseases, with both histological and clinical evidence of hepatitis [23]. Alcohol consumption was assessed through interviews with patients and, when available, their relatives. In the CHC subcohort, patients tested positive for anti-HCV for at least six months and had detectable HCV-RNA levels via RT-PCR. In the CHB subcohort, infection was diagnosed based on persistent positivity for HBsAg for over six months. Among these patients, 12 (16.7%) were positive for HBeAg, while 54 (83.3%) were negative.

The study excluded patients with autoimmune hepatitis, primary biliary cholangitis, significant alcohol consumption, HIV co-infection, dual infections (HBV/HCV or HBV/HDV), and suspected/confirmed liver cancer.

Clinical and laboratory assessments were conducted as part of routine pre-biopsy evaluations. Laboratory investigations, encompassing complete blood count, clinical chemistry panels, and coagulation profiles, were performed at a median interval of 2 days (IQR 0–10) prior to liver biopsy. When indicated, coagulation tests and platelet counts were repeated immediately before the procedure. Physical examinations, including measurements of body weight and height, were carried out within 24 h preceding the biopsy; body mass index (BMI) was calculated using the standard formula: BMI = body weight [kg]/(height [m])^2^.

**Figure 1:**
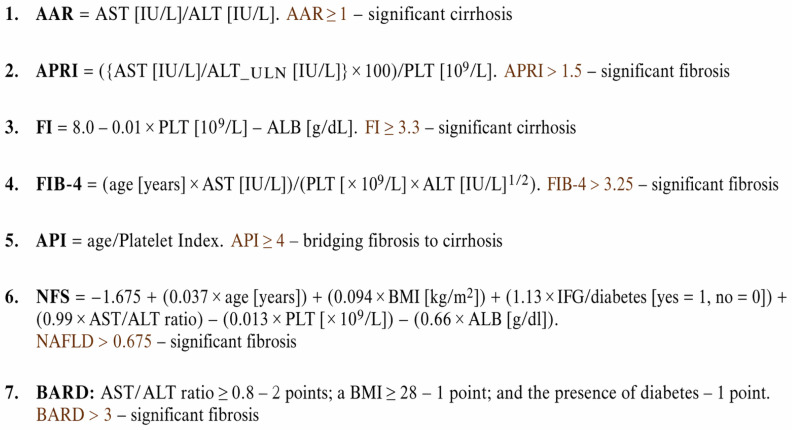
Presentation of formulas used to calculate non-invasive fibrosis assessment scores.

The liver fibrosis stage was non-invasively assessed using a combination of biochemical and radiological markers. Biochemical indices included AAR, APRI, FI, FIB-4, and API scores. In addition, TE was performed as a radiological method for fibrosis estimation. The formulas used to calculate these indices are presented in figure (Figure 1) [24]. All non-invasive assessments were performed immediately prior to liver biopsy.

Liver biopsies were performed using modified Menghini needles (1.4 or 1.6 mm in diameter). Specimens containing at least eight portal tracts and a minimum length of 15 mm were considered adequate for histological analysis. Samples were fixed in formalin, paraffin-embedded, and stained with hematoxylin–eosin and Masson’s trichrome. All histological evaluations were conducted by experienced pathologists from a single university center (Institute for Pathology, Faculty of Medicine, University of Belgrade), all of whom were blinded to laboratory and elastography findings. In patients with CVH, liver fibrosis was graded according to the Batts–Ludwig scoring system (F0–F4), where F0 indicates absence of fibrosis, F1 represents mild fibrosis, F2 represents moderate fibrosis, F3 represents severe fibrosis without cirrhosis, and F4 denotes cirrhosis (24). In patients diagnosed with MASH, the NAFLD Activity Score (NAS) was utilized to sum of steatosis, lobular inflammation, and ballooning (range 0–8) [25].

The TE was performed using a FibroScan^®^ device (EchoSens, Paris, France) with M probe. All TE assessments were performed by an experienced operator blinded to histological data. Ten valid measurements were obtained per patient, and the median liver stiffness (LS) value in kilopascals (kPa) was recorded. Measurements were considered valid if the success rate was ≥60% and the interquartile range (IQR) was <20% of the median. TE was performed within a median of 3 days (IQR 0–7) before liver biopsy. Stages of liver fibrosis were classified based on FibroScan^®^ scores as follows: F0–F1 (<7 kPa), F2 (7–8.99 kPa), F3 (9–12.49 kPa), and F4 or cirrhosis (12.50 kPa). Due to the gradual introduction of TE into routine clinical practice in Serbia starting in 2019, FibroScan^®^ was performed in only 21% of patients in the MASH cohort and in 87.5% of those in the CVH cohort. Additionally, during the early study period, the lack of XL probes limited the applicability of TE in patients with a BMI greater than 30, further reducing its use among individuals with MASH, who more frequently exhibited obesity. Consequently, TE data were not analyzed for the MASH group in this study.

Data were analyzed using SPSS Statistics for Windows, version 22. The Kolmogorov–Smirnov test assessed distribution normality. For normally distributed continuous variables, independent samples *t*-tests were employed; otherwise, the non-parametric Mann–Whitney U test was applied. Categorical variables were evaluated using Fisher’s exact test and the Chi-square test. Receiver Operating Characteristic (ROC) curves were constructed, and the area under these curves, along with their corresponding cut-off points, sensitivities, and specificities, were determined to assess the diagnostic accuracy of serum fibrosis indices. Given the potential confounding effect of alcohol use on fibrosis progression, a sensitivity analysis was performed to assess its impact on the study results.

The study was conducted in accordance with ethical standards outlined in the Declaration of Helsinki. This retrospective study was approved by the Ethics Committee of the University Clinical Center of Serbia no 1051/20. As part of the standard hospital admission procedure, patients signed a consent form for all diagnostic and therapeutic procedures. This consent also included permission to analyze patients’ medical records for research purposes.

## 3. Results

A total of 237 patients were enrolled in the study, including 77 individuals (32.5%) diagnosed with MASH and 160 (67.5%) with CVH. The mean age of the overall population was 51.27 ± 14.29 years. Within both cohorts, no statistically significant age differences were observed between male and female participants (*p* > 0.05). Patients in the CVH cohort were significantly older (*p* < 0.001) and had a significantly higher proportion of male individuals (*p* < 0.001) compared to the MASH cohort. In contrast, metabolic syndrome was significantly more prevalent among patients in the MASH cohort prior to the diagnosis of liver disease (*p* < 0.001) (Table 1).

Regarding fibrosis staging, cirrhosis was significantly more common among CVH patients (*p* < 0.001), while the absence of fibrosis was more frequently observed in those with MASH (*p* < 0.001). Reflecting the more advanced stage of liver disease, the CVH cohort demonstrated significantly higher values across all evaluated non-invasive fibrosis scores, including a higher median liver stiffness measurement—LSM (*p* < 0.001), elevated median serum alpha-fetoprotein levels (*p* < 0.01), and lower median serum albumin concentrations (*p* = 0.014) and platelet counts (*p* = 0.001) (Table 1).

Among the 160 patients with CVH, 94 (58.7%) were classified into the CHB subcohort, while 66 (41.2%) comprised the CHC subcohort. No significant sex-based age differences were observed within either subcohort (*p* > 0.05). Patients in the CHB group were significantly older (*p* < 0.001) and more likely to be male (*p* = 0.004) than those in the CHC subcohort. Histological analysis revealed that the absence of fibrosis (*p* < 0.001) and mild fibrosis (*p* = 0.015) were significantly more frequent among CHB patients. A borderline statistically significant difference was noted for cirrhosis, with a trend toward higher prevalence in the CHC subcohort (Table 2).

### 3.1. Diagnostic Performance of Non-Invasive Methods for F4 Stage Fibrosis in the MASH Cohort

**Figure 2:**
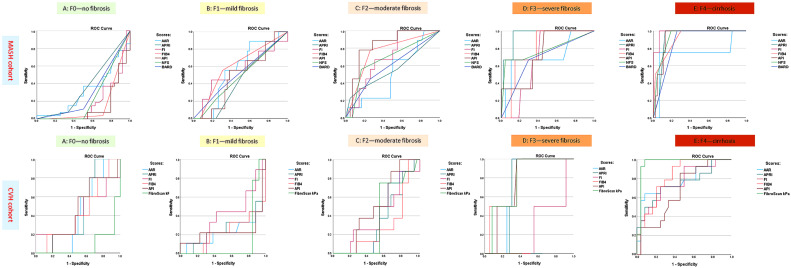
Presentation of ROC curves evaluating non-invasive approaches for fibrosis assessment across the examined cohorts: NASH cohort and HCV cohort.

Statistically significant high AUROC values for APRI, FIB-4, NFS, and BARD indicate excellent performance of these tests in identifying cirrhosis (*p* < 0.05). The AUROC values for APRI, FIB-4, NFS, and BARD for detecting cirrhosis were 0.878 (95% confidence interval [CI]: 0.841–0.902), 0.915 (95% CI: 0.892–0.934), 0.931 (95% CI: 0.902–0.960), and 0.872 (95% CI: 0.861–0.891), respectively. The threshold values for each of these scores were calculated. Specifically, patients with an APRI score above 1.410 have a 71.6% likelihood of confirming cirrhosis upon pathological examination, while patients with a FIB-4 score greater than 2.50 have a 71.9% chance of cirrhosis confirmation upon histological analysis. Additionally, patients with an NFS score above 1.12 have an 80.9% probability of cirrhosis confirmation, and patients with a BARD score exceeding 2.5 have a 78.5% chance of confirming cirrhosis upon histological analysis. In contrast, significantly lower AUROC values were observed for stages F0–3, which corresponded to reduced sensitivity and specificity of the scores in predicting these stages. The performance of these scores in ROC analysis is shown in Table 3 and Figure 1 and Figure 2.

### 3.2. Diagnostic Performance of Non-Invasive Methods for F4 Stage Fibrosis in the CVH Cohort

**Figure 3:**
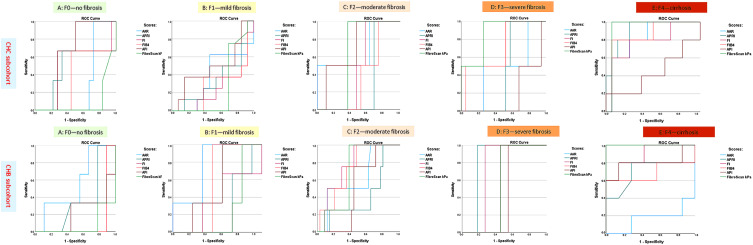
Presentation of ROC curves evaluating non-invasive approaches for fibrosis assessment across the examined cohorts: CHB and CHC subcohorts.

Statistically significant high AUROC values for APRI, FIB-4, and LSM by FibroScan^®^ indicate excellent performance of these tests in identifying cirrhosis in the CHC subcohort (*p* < 0.05). The AUROC values for APRI, FIB-4, and LSM by FibroScan^®^ for detecting cirrhosis were 0.931 (95% confidence interval [CI]: 0.910–0.953), 0.863 (95% CI: 0.838–0.887), and 0.938 (95% CI: 0.914–0.968), respectively. The threshold values for each of these scores were calculated. Specifically, patients with an APRI score above 1.54 have a 79.8% likelihood of confirming cirrhosis upon pathological examination, while patients with a FIB-4 score greater than 3.12 have an 81% chance of cirrhosis confirmation upon histological analysis. Additionally, patients with a LSM by FibroScan^®^ score above 12.2 kPa have a 94.5% probability of cirrhosis confirmation upon histological analysis. In contrast, significantly lower AUROC values were observed for stages F0–3, which corresponded to reduced sensitivity and specificity of the scores in predicting these stages. The performance of these scores in ROC analysis is shown in Table 3 and Figure 2 and Figure 3.

Statistically significant high AUROC values for FIB-4 and LSM by FibroScan^®^ indicate excellent performance of these tests in identifying cirrhosis in the CHB subcohort (*p* < 0.05). The AUROC values for FIB-4 and LSM by FibroScan^®^ for detecting cirrhosis were 0.821 (95% CI 0.802–0.940) and 0.987 (95% CI 0.978–0.996), respectively. The threshold values for each of these scores were calculated. Specifically, patients with a FIB-4 score greater than 2.89 have a 79.7% probability of cirrhosis confirmation upon pathological examination, while patients with an LSM by FibroScan^®^ score above 11.6 kPa have a 98.2% probability of cirrhosis confirmation upon histological examination. The performance of these scores in ROC analysis is shown in Table 3 and Figure 2. In contrast, significantly lower AUROC values were observed for stages F0–3, which corresponded to reduced sensitivity and specificity of the scores in predicting these stages. The performance of these scores in ROC analysis is shown in Table 4 and Figure 1 and Figure 2

The sensitivity analysis did not materially change the identified predictors, confirming the robustness of the primary findings; detailed results are provided in Supplementary Table S1.

## 4. Discussion

This study analyzes the diagnostic performance of non-invasive methods, including APRI, FIB-4, and TE, in detecting advanced liver fibrosis (F4 stage) in patients with MASH and CHV. The results demonstrated that non-invasive tests such as APRI, FIB-4, and TE performed well in identifying cirrhosis in the CVH cohort, with high sensitivity and specificity, particularly in the CHC subcohort. However, these methods were less effective in the MASH cohort, where the diagnostic performance was generally lower but still good, with particular emphasis on the importance of specifically tailored NFS and BARD scores for this indication. Furthermore, FibroScan^®^ demonstrated high diagnostic accuracy for cirrhosis in the CVH cohort; however, these findings should be interpreted with caution given the limited availability of TE in the MASH group.

Our study indicates that the highest AUROC for predicting cirrhosis in patients with MASH is observed with the NFS, followed by FIB-4, then BARD, and finally APRI. The other analyzed scores did not demonstrate statistically significant predictive power. In the study by Angulo et al., which validated the NFS score, using the higher cut-off value of 0.676 allowed for a highly accurate diagnosis of advanced fibrosis—positive predictive value was 90% in the estimation group and 82% in the validation group [26]. This model would have enabled clinicians to avoid liver biopsy in about 75% of patients, with correct prediction in 90% of those cases [26]. Our findings on predictive performance are in agreement with those reported in both the original study by the score developers and the subsequent validation study [26,27]. In our cohort, the application of this model suggests a high potential for reducing the need for liver biopsy; however, these findings should be interpreted cautiously given the study design and cohort characteristics.

The FIB-4 score is not designed to differentiate between simple steatosis and steatohepatitis and therefore should not be used for diagnosing MASH [28]. The focus on cirrhosis (F4) was chosen due to its major prognostic and therapeutic implications, including the need for surveillance and management of complications, although we acknowledge that earlier fibrosis stages are also clinically relevant. Its potential utility lies in estimating the likelihood of advanced fibrosis in individuals with suspected MASLD [29]. While some studies have aimed to distinguish patients with minimal fibrosis (stage 0–1) from those with more advanced stages, our approach—consistent with previous research—focused on identifying patients with stage 4 fibrosis, where FIB-4 demonstrated high sensitivity and specificity, suggesting its potential utility in clinical practice, particularly for identifying advanced fibrosis [30,31]. In the study conducted by Shah A et al., the AUROC for FIB-4 in diagnosing advanced fibrosis was 0.8, outperforming other non-invasive panels tested, although slightly lower than the AUROC of 0.9 reported for the LFM score [31].

This study demonstrated that the APRI score has a statistically significant AUROC (0.878) for predicting F4 fibrosis in the MASH cohort, with a cut-off value of 1.41. A related study found that the ROC for APRI was 0.85, with optimal cut-off values at 0.98, resulting in a sensitivity of 75% and specificity of 86% for predicting advanced fibrosis (>F3). A meta-analysis including 13 studies that evaluated the predictive power of APRI for cirrhosis development indicated that the APRI score had the lowest predictive value compared to FIB-4, NFS, and BARD [32].

Our study suggests that a BARD score greater than 2.5 predicts cirrhosis with a sensitivity of 78.5%. In the validation study of the BARD score for evaluating MASH, based on the results of logistic regression analysis with forced entry, three variables were combined into a weighted sum (BMI ≥ 28 = 1 point, AAR ≥ 0.8 = 2 points, DM = 1 point) to form an easily calculated composite score for predicting advanced fibrosis, known as the BARD score [33]. A score between 2 and 4 was associated with an odds ratio of 17 (95% CI: 9.2 to 31.9) for advanced fibrosis and a negative predictive value of 96% [33]. An Argentine study reports a significantly lower predictive value of the BARD score in predicting cirrhosis, with sensitivity of 51.4%, specificity of 77.2%, NPV of 81.3%, and PPV of 45.2% [34]. The AUROC values were 0.68 (95% CI: 0.57–0.78) for MASLD fibrosis and 0.67 (95% CI: 0.56–0.77) for BARD scores, respectively.

In this single-center evaluation of non-invasive liver fibrosis assessment methods, we observed that in the CHB subgroup, both FIB-4 and LSM via FibroScan^®^ showed significant AUROC, with FibroScan^®^ demonstrating a higher AUROC. In our study, a FibroScan^®^ LSM cut-off value of 11.6 kPa was found to have statistically significant predictive value for cirrhosis, with a sensitivity of 91.2% and specificity of 85.5%. These findings are in complete agreement with the largest meta-analysis of this type currently known to the authors [35]. In the meta-analysis, a total of 18 studies with 2772 patients were analyzed. The mean AUROC for cirrhosis (F4) was 0.929 (95% CI, 0.928–0.929). The cut-off value for F4 was 11.7 kPa (range, 7.3–17.5), with a sensitivity of 84.6% and specificity of 81.5% [35]. A related study that compared laboratory scores with LSM FibroScan^®^ found that FIB-4 had the best performance for predicting cirrhosis, with an AUROC of 0.851 in HBsAg-positive patients [22]. While there is no specific threshold for detecting cirrhosis using FIB-4 according to WHO guidelines, a study by these authors suggests an upper cut-off value of 4 for cirrhosis detection, derived from the ROC curve coordinates [8,9,10,22]. This cut-off value demonstrated 80% sensitivity, 91.7% negative predictive value (NPV), 90.8% specificity, and 60.5% positive predictive value (PPV) [22]. Additionally, a study validating the use of FIB-4 in CHB patients reported that a score above 3.6 had a 93.2% NPV and 90.8% PPV for detecting cirrhosis [36]. These authors also suggest that using FIB-4 in this indication could eliminate the need for biopsy in approximately 70% of cases [36].

In the DAA era, where nearly all patients with CHC can achieve sustained virologic response (SVR), the clinical priority has shifted from identifying early-stage fibrosis (F0–F1) to reliably detecting advanced fibrosis and cirrhosis (F3–F4) [22]. This study demonstrated that FIB-4 was superior to APRI in identifying patients with advanced fibrosis or cirrhosis in the setting of CHC (AUC of 0.905 for FIB-4 versus 0.831 for APRI). Similar findings have been reported in other studies as well [16,37,38]. In this study, LMF demonstrated the highest AUROC (0.938), with a cut-off value of 12.2 for predicting cirrhosis. Multiple studies have consistently recognized LMF as the gold standard for non-invasive assessment of advanced fibrosis, and our findings are fully in line with this established evidence [38,39,40,41]. In our experience, the application of TE (FibroScan^®^) with a cut-off value of 12.2 kPa in the CHC cohort enabled the avoidance of liver biopsy in 92 out of 94 patients (97.9%), with correct classification in all cases (100%). Specifically, all 34 patients with cirrhosis were accurately identified, and 58 out of 60 non-cirrhotic patients were correctly classified, reflecting a high sensitivity (99.8%) and specificity (96.5%). These findings highlight the strong clinical utility of FibroScan^®^ as a reliable non-invasive alternative to liver biopsy in this patient population.

Beyond diagnostic accuracy, our findings also support the clinical utility of non-invasive tools, particularly transient elastography, due to their reproducibility and suitability for longitudinal assessment. In line with previous studies, liver stiffness dynamics over time may provide additional insight into fibrosis regression or progression, especially in the post-treatment setting [42].

This retrospective, single-center study has several limitations, including inability to control for confounding variables and potential selection bias, limited external validity, unavailability of FibroScan^®^ for some MASH patients due to its later introduction and lack of XL probes, uneven representation of etiological groups with a dominance of viral hepatitis, a limited number of cirrhotic patients in the MASH cohort, and exclusion of patients with certain comorbidities, which reduces the generalizability of the findings to the broader population. Importantly, the high diagnostic performance observed in this study may be overestimated due to the single-center design, potential spectrum bias with enrichment of advanced disease, and the absence of external validation.

## 5. Conclusions

In conclusion, this study highlights those non-invasive methods for assessing advanced fibrosis, such as LMF, FIB4, NFS, BARD, and APRI, show excellent performance and are potentially very useful for the assessment of liver fibrosis in patients with CVH and MASH. These findings support current recommendations advocating for the use of non-invasive tools, particularly in resource-limited settings, where access to more advanced diagnostics may be constrained. Among these, LMF demonstrated the best performance in determining cirrhosis. However, liver biopsy remains the gold standard and is still necessary in certain indications. A broader validation of these results is essential to confirm their reliability and applicability in diverse clinical settings.

## Figures and Tables

**Table 1:** Presentation of sociodemographic, clinical, and laboratory parameters of the observed cohort.

Variable		MASH Cohort N = 77 (32.5%)	CVH Cohort N = 160 (67.5%)	*p*
Gender	Male	62 (80.5%)	99 (61.9%)	**0.004**
	Female	15 (19.5%)	61 (38.1%)	
Age		45.75 ±9.91	54.48 ±13.17	**0.001**
Chronic viral hepatitis	CVH B	n/a	66 (41.2%)	n/a
	CVH C		94 (58.7%)	
Alcohol use disorder		2 (2.59%)	69 (43.12%)	**0.001**
Body mass index		29.31 ± 6.12	26.38 ±5.99	0.125
Metabolic syndrome		61 (79.22%)	57 (35.62%)	**0.001**
Fibrosis stage—histopathological				
F0—No fibrosis		39 (50.6%)	20 (12.5%)	**0.001**
F1—Mild fibrosis		16 (20.8%)	33 (20.6%)	0.874
F2—Moderate fibrosis		12 (15.6%)	32 (20.0%)	0.264
F3—Severe fibrosis		4 (5.2%)	15 (9.38%)	0.174
F4—Cirrhosis		6 (7.8%)	59 (36.9%)	**0.001**
Laboratory parameters, med. (IQR)				
Platelets [10^9^/L]		273.5 (205.0–315.0)	152.5 (105.5–195.0)	**0.001**
Alanine aminotransferase [U/L]		87.5 (25.0–105.5)	81.5 (30.5–99.5)	0.215
Aspartate aminotransferase [U/L]		55.0 (39.0–88.5)	53.0 (12.5–125.8)	0.154
Gamma-glutamyl transferase [U/L]		35.5 (12.0–61.0)	39.5 (10.5–54.0)	0.586
International normalized ratio		0.95 (0.87–1.05)	0.87 (0.74–1.0)	0.094
Albumin, med. [g/L]		45.0 (34.5–49.5)	38.0 (30.5–42.0)	**0.014**
Alpha-fetoprotein [μg/L]		3.1 (0.5–5.0)	6.5 (0.5–8.0)	**0.001**
Fibrosis assessment score				
ARR score		0.64 (0.51–0.71)	0.79 (0.57–1.08)	**0.001**
APRI score		0.65 (0.50–0.78)	0.95 (0.60–1.25)	**0.001**
FI score		1.37 (0.90–1.93)	2.42 (1.70–4.70)	**0.001**
FIB-4 score		1.18 (0.81–1.65)	1.56 (1.04–3.97)	**0.001**
API score		0.21 (0.14–0.26)	1.30 (1.21–1.42)	**0.001**
NFS score		1.05 (0.50–1.50)	n/a	
BARD score		1.25 (0.55–1.75)		
FibroScan—kPa		5.50 (3.0–7.5)	6.72 (5.20–8.21)	**0.001**

Legend: Bolded values are statistically significant.

**Table 2:** Presentation of sociodemographic, clinical, and laboratory parameters of the observed subcohort.

Variable		CHC Subcohort N = 94 (58.7%)	CHB Subcohort N = 66 (41.2%)	*p*
Gender	Male	51 (54.2%)	48 (72.7%)	**0.004**
	Female	43 (45.7%)	18 (27.8%)	
Age		51.23 ±10.26	55.97 ±9.24	**0.001**
Alcohol use disorder		42 (44.7%)	27 (40.91%)	**0.001**
Body mass index		25.19 ± 4.27	26.91 ±4.38	0.241
Metabolic syndrome		22 (23.4%)	35 (53.0%)	**0.001**
Fibrosis stage—histopathological				
F0—No fibrosis		3 (3.2%)	17 (25.8%)	**0.001**
F1—Mild fibrosis		26 (30.8%)	7 (10.6%)	**0.015**
F2—Moderate fibrosis		16 (17.0%)	16 (24.2%)	0.241
F3—Severe fibrosis		11 (11.7%)	4 (6.1%)	0.174
F4—Cirrhosis		38 (40.4%)	21 (31.8%)	0.087
Laboratory parameters, med. (IQR)				
Platelets [10^9^/L]		131.2 (101.2–184.0)	159.5 (114.5–199.5)	0.106
Alanine aminotransferase [U/L]		72.0 (26.5–94.3)	89.8 (32.5–100.5)	0.069
Aspartate aminotransferase [U/L]		56.0 (16.0–95.7)	50.5 (10.5–106.7)	0.577
Gamma-glutamyl transferase [U/L]		35.5 (11.5–62.0)	40.9 (12.0–62.0)	0.614
International normalized ratio		0.79 (0.73–0.98)	0.88 (0.72–1.1)	0.381
Albumin, med. [g/L]		39.5 (31.0–43.0)	42.3 (34.5–46.0)	0.254
Alpha-fetoprotein [μg/L]		7.1 (0.5–9.0)	6.1 (0.5–7.5)	0.677
Fibrosis assessment score				
ARR score		0.74 (0.52–1.01)	0.82 (0.64–1.09)	0.367
APRI score		0.85 (0.62–1.21)	0.93 (0.72–1.39)	0.364
FI score		1.95 (1.64–2.70)	1.86 (1.50–2.70)	0.498
FIB-4 score		1.13 (1.01–1.58)	1.63 (1.02–1.45)	0.277
API score		1.35 (1.20–1.48)	1.36 (1.22–1.44)	0.982
FibroScan—kPa		6.90 (6.1–7.8)	6.35 (5.85–6.60)	0.089
CHB—phases of infection				
HBeAg + chronic infection		n/a	11 (16.7%)	n/a
HBeAg + chronic hepatitis			9 (13.6%)	
HBeAg—chronic infection			33 (50%)	
HBeAg—chronic hepatitis			13 (19.7%)	
HCV—genotype				
G1a		34 (36.1%)	n/a	
G1b		17 (18.1%)		
G2		3 (3.2%)		
G3		31 (33%)		
G4		9 (9.6%)		

Legend: Bolded values are statistically significant.

**Table 3:** Parameters on the Receiver Operating Characteristics curve analysis.

Variable	MASH Cohort		CVH Cohort	
ARR score	Area under the curve	0.742	Area under the curve	0.778
	Standard error	0.172	Standard error	0.083
	Cut-off	1.005	Cut-off	1.06
	Sensitivity	55.3%	Sensitivity	64.3%
	Specificity	78.6%	Specificity	81.4%
	*p*	0.111	*p*	0.097
APRI score	Area under the curve	0.878	Area under the curve	0.747
	Standard error	0.053	Standard error	0.088
	Cut-off	1.410	Cut-off	1.39
	Sensitivity	71.6%	Sensitivity	71.4%
	Specificity	83.9%	Specificity	67.3%
	*p*	**0.013**	*p*	0.097
FI score	Area under the curve	0.984	Area under the curve	0.753
	Standard error	0.018	Standard error	0.089
	Cut-off	3.235	Cut-off	2.42
	Sensitivity	83.9%	Sensitivity	64.3%
	Specificity	93.6%	Specificity	75.5%
	*p*	0.061	*p*	**0.019**
FIB-4 score	Area under the curve	0.915	Area under the curve	0.821
	Standard error	0.053	Standard error	0.088
	Cut-off	2.50	Cut-off	2.69
	Sensitivity	71.9%	Sensitivity	71.4%
	Specificity	80.9%	Specificity	71.8%
	*p*	**0.016**	*p*	**0.016**
API score	Area under the curve	0.926	Area under the curve	0.674
	Standard error	0.039	Standard error	0.088
	Cut-off	2.265	Cut-off	2.165
	Sensitivity	78.5%	Sensitivity	71.4%
	Specificity	92.6%	Specificity	85.2%
	*p*	0.075	*p*	0.077
NFS score	Area under the curve	0.931	n/a	
	Standard error	0.041		
	Cut-off	1.12		
	Sensitivity	80.9%		
	Specificity	86.1%		
	*p*	**0.015**		
BARD score	Area under the curve	0.872	n/a	
	Standard error	0.055		
	Cut-off	2.5		
	Sensitivity	78.5%		
	Specificity	82.3%		
	*p*	**0.014**		

Legend: Bolded values are statistically significant.

**Table 4:** Parameters on the Receiver Operating Characteristics curve analysis.

Variable	CHC Subcohort		CHB Subcohort	
ARR score	Area under the curve	0.740	Area under the curve	0.171
	Standard error	0.097	Standard error	0.138
	Cut-off	1.045	Cut-off	1.06
	Sensitivity	80.0%	Sensitivity	70.6%
	Specificity	75.0%	Specificity	76.8%
	*p*	0.069	*p*	0.625
APRI score	Area under the curve	0.931	Area under the curve	0.747
	Standard error	0.064	Standard error	0.088
	Cut-off	1.540	Cut-off	1.39
	Sensitivity	79.8%	Sensitivity	76.9%
	Specificity	76.5%	Specificity	78.2%
	*p*	**0.006**	*p*	0.097
FI score	Area under the curve	0.775	Area under the curve	0.753
	Standard error	0.127	Standard error	0.089
	Cut-off	3.250	Cut-off	2.42
	Sensitivity	73.9%	Sensitivity	72.8%
	Specificity	80.3%	Specificity	79.4%
	*p*	0.069	*p*	**0.019**
FIB-4 score	Area under the curve	0.863	Area under the curve	0.821
	Standard error	0.098	Standard error	0.088
	Cut-off	3.120	Cut-off	2.89
	Sensitivity	81.0%	Sensitivity	79.7%
	Specificity	87.5%	Specificity	76.9%
	*p*	0.017	*p*	**0.016**
API score	Area under the curve	0.438	Area under the curve	0.674
	Standard error	0.169	Standard error	0.088
	Cut-off	2.260	Cut-off	2.165
	Sensitivity	70.1%	Sensitivity	71.6%
	Specificity	64.6%	Specificity	68.2%
	*p*	0.680	*p*	0.077
FibroScan—kPa	Area under the curve	0.938	Area under the curve	0.987
	Standard error	0.061	Standard error	0.083
	Cut-off	12.20	Cut-off	11.6%
	Sensitivity	94.5%	Sensitivity	98.2%
	Specificity	96.5%	Specificity	94.5%
	*p*	**0.004**	*p*	**0.002**

Legend: Bolded values are statistically significant.

## Data Availability

The data supporting the findings of this study are not publicly available due to privacy and ethical restrictions related to the protection of patient personal data. Access to the data may be considered upon reasonable request to the corresponding author, subject to approval by the relevant institutional ethics committee.

## Supplementary Materials

The following supporting information can be downloaded at: https://www.mdpi.com/article/10.3390/biomedicines14050988/s1, Table S1. Receiver Operating Characteristic (ROC) Analysis Following Exclusion of Patients with Alcohol Use Disorder.
