# Supplementary material for: Cut or Count? Evaluating Advanced Fibrosis Assessment Tools in MASH and Chronic Viral Hepatitis
Source: Biomedicines. 2026 Apr 25;14(5):988. doi: 10.3390/biomedicines14050988 (PMC13203587; doi:10.3390/biomedicines14050988)
Supplement: Supplementary file 1 [file biomedicines-14-00988-s001.zip › biomedicines-4233408-supplementary.pdf]

Supplementary Table S1. Receiver Operating Characteristic (ROC) Analysis Following Exclusion of Patients with Alcohol Use Disorder

Variable	MASH cohort		CVH cohort	
ARR score	Area under the curve	0.695	Area under the curve	0.762
	Standard error	0.162	Standard error	0.079
	Cut-off	0.98	Cut-off	1.08
	Sensitivity	56.8%	Sensitivity	66.1%
	Specificity	76.2%	Specificity	79.8%
	p	0.214	*p*	0.082
APRI score	Area under the curve	0.861	Area under the curve	0.732
	Standard error	0.057	Standard error	0.091
	Cut-off	1.360	Cut-off	1.420
	Sensitivity	69.4%	Sensitivity	70.2%
	Specificity	81.7%	Specificity	65.9%
	p	**0.018**	*p*	0.104
FI score	Area under the curve	0.972	Area under the curve	0.741
	Standard error	0.021	Standard error	0.092
	Cut-off	3.102	Cut-off	2.35
	Sensitivity	82.1%	Sensitivity	62.8%
	Specificity	91.4%	Specificity	73.6%
	p	0.068	p	**0.023**
FIB-4 score	Area under the curve	0.902	Area under the curve	0.741
	Standard error	0.057	Standard error	0.092
	Cut-off	2.42	Cut-off	2.35
	Sensitivity	70.3%	Sensitivity	62.8%
	Specificity	78.6%	Specificity	73.6%
	p	**0.019**	*p*	**0.018**
API score	Area under the curve	0.911	Area under the curve	0.662
	Standard error	0.043	Standard error	0.091
	Cut-off	2.18	Cut-off	2.10
	Sensitivity	76.4%	Sensitivity	69.8%
	Specificity	90.3%	Specificity	83.7%
	p	0.082	*p*	0.091
NFS score	Area under the curve	0.918	n/a	
	Standard error	0.045		
	Cut-off	1.05		
	Sensitivity	79.2%		
	Specificity	84.3%		
	p	**0.017**		
BARD score	Area under the curve	0.859	n/a	
	Standard error	0.059		
	Cut-off	2.4		
	Sensitivity	76.8%		
	Specificity	80.5%		
	p	**0.019**		
